# Inflammation and Immune-Related Candidate Gene Associations with Acute Lung Injury Susceptibility and Severity: A Validation Study

**DOI:** 10.1371/journal.pone.0051104

**Published:** 2012-12-14

**Authors:** D. Shane O'Mahony, Bradford J. Glavan, Tarah D. Holden, Christie Fong, R. Anthony Black, Gail Rona, Paula Tejera, David C. Christiani, Mark M. Wurfel

**Affiliations:** 1 Division of Pulmonary and Critical Care Medicine, Harborview Medical Center, University of Washington, Seattle, Washington, United States of America; 2 Harvard School of Public Health, Harvard University, Boston, Massachusetts, United States of America; 3 Institute of Translational Health Sciences (ITHS), University of Washington, Seattle, Washington, United States of America; Kunming Institute of Zoology, Chinese Academy of Sciences, China

## Abstract

**Introduction:**

Common variants in genes related to inflammation, innate immunity, epithelial cell function, and angiogenesis have been reported to be associated with risks for Acute Lung Injury (ALI) and related outcomes. We tested whether previously-reported associations can be validated in an independent cohort at risk for ALI.

**Methods:**

We identified 37 genetic variants in 27 genes previously associated with ALI and related outcomes. We prepared allelic discrimination assays for 12 SNPs from 11 genes with MAF>0.05 and genotyped these SNPs in Caucasian subjects from a cohort of critically ill patients meeting criteria for the systemic inflammatory response syndrome (SIRS) followed for development of ALI, duration of mechanical ventilation, and in-hospital death. We tested for associations using additive and recessive genetic models.

**Results:**

Among Caucasian subjects with SIRS (n = 750), we identified a nominal association between rs2069832 in *IL6* and ALI susceptibility (OR_adj_ 1.61; 95% confidence interval [CI], 1.04–2.48, *P* = 0.03). In a sensitivity analysis limiting ALI cases to those who qualified for the Acute Respiratory Distress Syndrome (ARDS), rs61330082 in *NAMPT* was nominally associated with risk for ARDS. In terms of ALI outcomes, SNPs in *MBL2* (rs1800450) and *IL8* (rs4073) were nominally associated with fewer ventilator-free days (VFDs), and SNPs in *NFE2L2* (rs6721961) and *NAMPT* (rs61330082) were nominally associated with 28-day mortality. The directions of effect for these nominal associations were in the same direction as previously reported but none of the associations survived correction for multiple hypothesis testing.

**Conclusion:**

Although our primary analyses failed to statistically validate prior associations, our results provide some support for associations between SNPs in *IL6 and NAMPT* and risk for development of lung injury and for SNPs in *IL8*, *MBL2, NFE2L2* and *NAMPT* with severity in ALI outcomes. These associations provide further evidence that genetic factors in genes related to immunity and inflammation contribute to ALI pathogenesis.

## Introduction

Acute Lung Injury (ALI) and it's more severe manifestation, Acute Respiratory Distress Syndrome (ARDS) are associated with high mortality rates among intensive care unit patients [1]–[4]. Despite multiple clinical trials, no pharmacological agent has been shown to improve ALI-related outcomes [5]. Failure of these trials is due, in part, to an incomplete understanding of the key biologic pathways involved in the development and persistence of ALI in humans. One approach to identification of the key biologic pathways in ALI pathogenesis is gene-association studies.

Candidate gene studies in ALI have focused on variation within pathways thought to be important in ALI pathogenesis including genes involved in inflammation, immunity, oxidation, coagulation, angiogenesis, cell growth, endothelial barrier, surfactant function, and transcription regulation [6]. However, only a subset of putative ALI risk alleles have been validated in independent populations [7]–[11]. Robust associations between candidate gene polymorphisms and ALI validated in independent populations could support the development of more accurate models predicting risk for ALI and provide additional rationale for novel therapeutic interventions.

In this study, we sought to determine whether previously-reported associations between variants in genes of immunity, inflammation, angiogenesis and oxidative stress and risk for development of ALI or related outcomes, are robust. We used a nested case-control study design in a prospective cohort of critically ill patients with SIRS followed for development of ALI. Secondary analyses looked at ALI related outcomes in a case-only design.

## Methods

### SNP selection

We searched PubMed (www.ncbi.nlm.nih.gov/pubmed/) for publications reporting associations between genetic variants and development of ALI and/or ALI related outcomes as of August 2010. A panel of ALI investigators (DSO, BJG, MMW) selected variants based on strength of prior studies, biological relevance, and HapMap [12] minor allele frequencies (MAF) of >0.05 in Caucasians. Variants were then further narrowed to those for which Taqman™ allelic discrimination assays could be designed or that were in high linkage disequilibrium (LD) with another SNP for which an assay could be prepared. As we were unable to design a Taqman assay for rs1800795, we used a surrogate SNP, rs2069832 (pairwise r^2^ = 0.97).

### Study population

The cohort used for this study has been previously described [13]. Briefly, patients admitted to the intensive care unit at Harborview Medical Center, Seattle, WA (Dec 2006–Dec 2010) with systemic inflammatory response syndrome (SIRS) were followed prospectively for development of ALI and related outcomes such as death and other organ dysfunction. Patients were categorized as having SIRS if they had three or more of the following concurrently within a 24 hour period; a) body temperature (<36°C or >38°C) b) HR>90/min, c) RR>20/min or were on the vent and had PCO2<32 mmHg, and d) WBC<4,000/mm^3^ or >12,000/mm^3^. Exclusion criteria included trauma, HIV or immunosuppression, neurological injury, current diagnosis of cancer, and presence of a “do not resuscitate” order. ALI cases were as defined as per the American-European Consensus Committee (AECC) criteria for ARDS; presence of a proximal risk factor for ALI, presence of a PaO2/FiO2 (ratio of partial pressure of oxygen in arterial blood/fraction of oxygen in gas delivered) <300, absence of overt congestive heart failure, and a chest radiograph with bilateral parenchymal opacities as adjudicated by three critical care physicians [13]. We further subclassified cases as ARDS if the PaO2/FiO2 <200. According to the recently published Berlin Definition for Acute Respiratory Distress Syndrome, our definition of ALI would equate to mild, moderate, or severe ARDS while our definition of ARDS would equate to moderate and severe ARDS [14]. At-risk patients who did not meet criteria for development of ALI during the ICU hospitalization were classified as control subjects. This study was approved by the University of Washington, Division of Human Subjects Research.

### Genotyping

Genomic DNA was extracted from whole blood samples using the Puregene DNA Isolation Kit (Qiagen, CA). All SNPs were genotyped using Taqman-based allelic discrimination assays, (Applied Biosystems, CA), on a multichannel microfluidics chip (Fluidigm, CA). Genotyping was carried out per the vendor's protocol in a blinded fashion.

### Statistical Analysis

We identified differences in demographic variables between ALI and control subjects using Fisher's exact test for categorical variables and Student's *t*-test for continuous variables. Observed genotype frequencies were compared with expected frequencies to test for deviations from Hardy-Weinberg equilibrium using Fisher's exact test. To reduce possible confounding from population-stratification and racial differences, all analyses were restricted to Caucasian subjects. As a screen for major population substructure, we performed principal component analysis (PCA) using SNP genotypes from 67 SNPs available on our samples and which were also publically available for HapMap 3 subjects [12]. None of the first 5 PCs associated with case/control status.

We used multivariate logistic regression to estimate the genotype-specific odds ratio (OR) and 95% confidence interval (CI) for ALI susceptibility. The genotype associations were analyzed in both additive and recessive models. Covariates were chosen *a priori* based on known effects on risk for ALI development and mortality and included age, sex, APACHE III score, comorbidities, smoking status and alcohol abuse. A two sided *P* value <0.05 was considered to be nominally significant in all analyses. To control for type I error from multiple hypotheses testing, we used a false discovery rate (FDR) approach setting a threshold of 0.1 as indicative of corrected significance. FDR was calculated separately for each association test using the total number of SNPs tested.

We conducted a sensitivity analysis restricting case and control subjects to avoid phenotype misclassification. We restricted cases of ALI to only those qualifying for moderate and severe ARDS by oxygenation criteria (PaO2/FiO2 <200) and we restricted at-risk controls to only subjects who did not have an episode of hypoxemia (PaO2/FiO2 <300).

In secondary analyses we tested for associations between genotypes and ventilator free days (VFDs) using linear regression and mortality within 28 days using logistic regression, adjusting for covariates as above.

In a post hoc analysis, and for SNPs with adequate genotyping data available in prior publications, we completed unadjusted meta-analyses for genotype associations with ALI risk or mortality in ALI subjects by combining our results with the published data.

We estimated that, under an additive model, our study would have adequate statistical power (1-β>0.8) to detect associations of moderate strength with ALI (relative risk (RR) >1.5) for SNPs with MAF>0.2. Assuming a recessive model, we would have adequate power to detect a strong association (RR greater than or equal to 3) with SNPs having a MAF>0.25. See Figure S2 for a description of power calculations and the range of power over varying models, allele frequencies and genotype effect strengths.

Statistical analyses were run using the R statistical package and SNP and Variation Suite 7™ (Golden Helix™, Bozeman, MT).

## Results

### Characteristics of the Study Population

**Figure 1:**
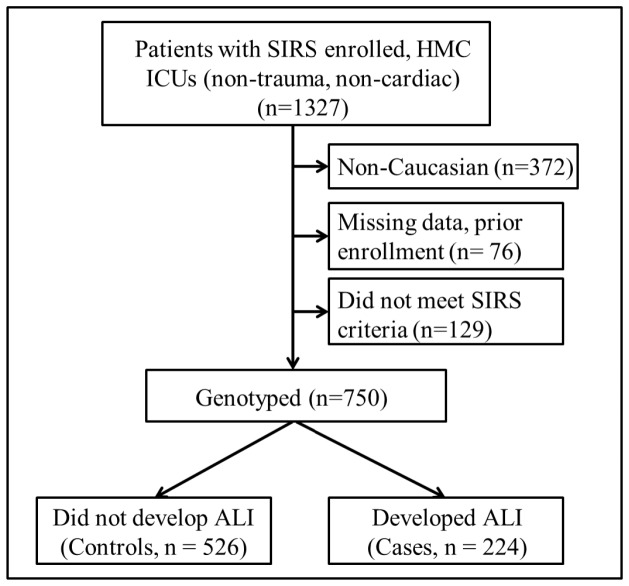
Flow diagram for Study design. Subjects with suspected systemic inflammatory response syndrome (SIRS), n = 1327, were enrolled at Harborview Medical Center (HMC), Seattle, WA during the period December 2006–December 2010. In this study, we excluded non-Caucasian subjects, subjects who were found to have previous enrollment or missing data, and subjects that did not meet 3 SIRS criteria present concurrently within a 24 hour period.

We genotyped 879 subjects; 238 subjects who developed ALI and 641 at-risk control subjects (See Figure 1 for the subject exclusion flow chart diagram). For analyses, we restricted only to subjects that met 3 criteria for SIRS (Figure 1). These subjects had a mean age of 54 (StdDev ±16) years, were predominantly male (63.1%), and were moderately ill with a mean APACHEIII score of 64.7 (StdDev ±25). The baseline characteristics for the study population and comparisons of co-morbidities and clinical risk factors for ALI between groups are shown in Table 1. Compared with subjects who did not develop ALI, ALI cases were of similar age, more likely to be male, and had higher APACHE III scores. The proportion of subjects with coexisting end-stage renal disease, liver failure, alcohol abuse, and shock was greater in the ALI group. Hospital mortality for subjects with ALI was 24.6% which is similar to observed rates in recent ARDSNet studies [15], [16]. As would be expected, ALI cases were more likely to have acute kidney injury, sepsis, shock and fewer VFDs.

**Table 1:** HMC SIRS cohort population characteristics.

	*All subjects*	*ALI Cases*	*At risk Controls*	*P value*
Subjects n	750	224	526	
Age, yrs, (IQR)	54 (45–64)	55 (44–66)	54 (44–64)	0.381
Male (%)	480 (64)	159 (71)	321 (61)	0.007
Caucasian, %	100	100	100	
ARDS	126 (16.8)	126 (56.3)	0	
APACHE III*	64.7±25	79±25	63±24	<0.001
ALI risk factor				
Sepsis	533 (71)	195 (87)	342 (65)	<0.001
Pneumonia	180 (24)	87 (39)	89 (17)	<0.001
Aspiration	90 (12)	49 (22)	42 (8)	<0.001
Transfusion	16 (2.1)	8 (3.6)	8 (1.5)	0.096
Comorbidities				
Diabetes	203 (27)	56 (25)	142 (27)	0.65
ESRD	18 (2.4)	3 (1.3)	15 (2.9)	0.30
Liver failure	90 (10.2)	35 (14.7)	55 (8.6)	0.08
Alcohol	263 (35)	94 (42)	168 (32)	0.015
COPD	143 (19)	40 (18)	100 (19)	0.75
CHF	83 (11)	31 (14)	47 (9)	0.06
Smoking	450 (60)	146 (65)	300 (57)	0.062
Outcomes				
VFD	19.3±10	13±11	22±10	<0.001
Death	120 (16)	56 (25)	63 (12)	<0.001
AKI	632 (84)	204 (91)	428 (81)	<0.001
Hemodialysis	53 (7)	25 (11)	32 (6)	0.013

Data is presented as mean ± SD for continuous variables and n (%) for categorical variables. P values represent results of t test comparison for difference of means for continuous variables and Fisher's exact test for frequency counts for categorical variables. IQR, interquartile range; VFD, ventilator free days; AKI, presence of acute kidney injury by AKIN score >/ = 1 with lowest creatinine during hospitalization used as baseline; Death, subject death within 28 days of admission.

### SNPs selected for validation

We searched PubMed for gene association studies of ALI and ARDS susceptibility and related outcomes. At the time of the search (August, 2010), we identified 37 genetic variants in 27 genes including SNPs, insertion/deletions, and SNP haplotypes (Table S1). The set of SNPs selected for this validation study tagged unique linkage disequilibrium bins that have been associated with ALI risk and/or ALI related outcomes and were located in or near the candidate genes. See Table 2 for the list of SNPs used in this study.

**Table 2:** Genetic variants studied.

GENE	SNP	MAF*	Effect	Case**	Control**	Reference
*ANGPT2* (Angiopoietin-2)	rs2515475	0.14	↑ risk ARDS	EA 449	1080 at risk	Su et al, 2009 [33]
*EGF* (Epidermal growth factor)	rs4444903	0.39	↑ risk ARDS	EA 416	1052 at risk	Shue et al, 2009 [34]
*IL6* (Interleukin-6)	rs1800795	0.47	↑ mortality in ARDS	EA; 96	88 at risk; 174 healthy	Marshall et al, 2002 [17]
*IL6* (Interleukin-6)	rs1800795 haplotypes		↑ risk ARDS			[11], [29], [30]
*IL8* (Interleukin-8)	rs4073	0.40	↑ days on MV, ↑ IL8 levels	EA; 23	74 at risk	Hildebrand et al, 2007 [20]
*IL10* (Interleukin-10)	rs1800896	0.47	↑ risk ARDS	EA; 211	429 at risk	Gong et al, 2006 [35]
*MBL2* (Mannose-binding Lectin 2)	rs1800450	0.15	↑ risk ARDS	EA; 212	412 at risk	Gong et al, 2007 [36]
*NFE2L2* (Nuclear factor, erythroid-derived, 2-like 2)	rs6721961	0.06	↑ risk ARDS	EA,AA; 30	60, matched case control	Marzec et al, 2007 [8]
*NAMPT* (Pre-B cell Colony-enhancing factor 1)	rs59744560, rs61330082	0.16, 0.33	↑ risk ARDS; ↓ risk ARDS	EA; 87	100 at risk	Ye et al, 2005 [19]
*SFTPB* (Surfactant. protein B)	rs1130866	0.43	↑ risk ARDS	EA,AA; 12	390 at risk	Quasney et al, 2004 [37]
*TNF* (Tumor necrosis factor α)	rs1800629	0.17	↑ mortality in ARDS	EA 237	476 at risk	Gong et al, 2005 [21]
*VEGFA* (vascular endothelial growth factor A)	rs3025039	0.19	↑ risk ARDS	EA 117	137 at risk; 103 healthy	Medford et al, 2005 [38]

* MAF, minor allele frequency in the HapMap;

** CEU population; EA, European American; AA, African American; Number refers to number of subjects in group of the referenced study.

### Genotyping results

We achieved genotype call rates across all subjects of over 99% for each of the 12 SNPs. One of the SNPs, rs4444903, had genotype frequencies for the control subjects that deviated from predicted frequency under Hardy Weinberg equilibrium (HWE). The remaining SNPs had genotype results for the control subjects that were not significantly different from HWE predictions using the χ^2^ test.

We did not observe strong evidence of population stratification by PCA comparing 67 shared genotypes among our subjects and HapMap3 subjects. When plotting PC1 vs. PC2, our subjects clustered with HapMap CEU and TSI subjects but not with Asian or African clusters as expected. No subject outliers or associations between phenotype and eigenvalue were observed. See Figure S1.

### Association between SNPs and development of ALI

Genotype frequencies for control and case subjects are presented in Table 3. The A allele of rs4444903 in EGF was over-represented in cases but genotype frequencies for this SNP in controls deviated from HWE.

**Table 3:** Genotype frequencies in at-risk controls versus ALI cases.

Gene	SNP rs#	At Risk Controls (%) (n = 526)			?^2^ p-value for HWE in at-risk control	ALI cases (%) (n = 224)			Fisher's Exact test genotype freq. (p-value)^a^
*SFTPB*	rs1130866	TT 134 (25)	TC 257 (49)	CC 135 (26)	0.60	TT 57 (25)	TC 116 (52)	CC 51 (23)	0.66
*MBL2*	rs1800450	CC 370 (70)	CT 142 (27)	TT 14 (3)	0.93	CC 162 (72)	CT 56 (25)	TT 6 (3)	0.64
*TNF*	rs1800629	GG 383 (71)	GA 131 (25)	AA 12 (2)	0.84	GG 160 (71)	GA 56 (27)	AA 4 (2)	0.82
*IL10*	rs1800896	TT 144 (27)	TC 256 (49)	CC 126 (24)	0.56	TT 51 (23)	TC 114 (51)	CC 59 (26)	0.22
*IL6*	rs2069832	GG 173 (33)	GA 273 (52)	AA 80 (15)	0.10	GG 78 (35)	GA 99(44)	AA 47 (21)	0.49
*ANGPT2*	rs2515475	CC 410 (78)	CT 102 (19)	TT 14 (3)	0.016	CC 178 (79)	CT 42 (19)	TT 4 (2)	0.53
*VEGF*	rs3025039	CC 381 (72)	CT 133 (25)	TT 12 (2)	0.92	CC 168 (75)	CT 52 (23)	TT 4 (2)	0.44
*IL8*	rs4073	TT 163 (31)	TA 252 (48)	AA 110 (21)	0.49	TT 61 (27)	TA 108 (48)	AA 55 (25)	0.19
*EGF*	rs4444903	AA 143 (27)	AG 301 (57)	GG 81 (15)	0.0002	AA 91 (41)	93 (42)	40 (18)	0.041
*NAMPT*	rs59744560	GG 398 (76)	GT 114 (22)	TT 14 (3)	0.10	GG 159 (71)	GT 59 (26)	TT 6 (3)	0.25
*NAMPT*	rs61330082	CC 300 (57)	CT 185 (35)	TT 40 (8)	0.13	CC 141 (63)	CT 67 (30)	TT 16 (7)	0.21
*NFE2L2*	rs6721961	GG 409 (78)	GT 109 (21)	TT 8 (2)	0.81	GG 175 (78)	GT 44 (20)	TT 5 (2)	0.93

HWE Hardy-Weinberg equilibrium;

a p value for the Fisher's exact test comparing genotypes for at risk controls and ALI cases.

In analyses adjusted for age, gender, APACHEIII score, presence of diabetes mellitus, end stage renal disease, chronic alcohol use, cirrhosis and smoking status, we identified a nominal association between the A allele of rs2069832 in *IL6* by recessive modeling (OR_adj_ 1.61; 95% confidence interval [CI], 1.04–2.48, *P* = 0.03) (Table 4). The direction of effect and magnitude of the odds ratio result was consistent with the prior publication [17]. However, this association result did not meet our predetermined threshold for FDR (FDR<0.1). We did not observe any other significant associations with ALI susceptibility for any of the remaining SNPs in adjusted (Table 4) or unadjusted analyses (Table 3).

**Table 4:** Associations with ALI susceptibility.

		ADDITIVE				RECESSIVE			
	Predictor	Odds Ratio	OR Conf. Interval	P-Value	FDR	Odds Ratio	OR Conf. Interval	P-Value	FDR
*SFTPB*	rs1130866	0.93	0.73–1.18	0.55	0.72	0.83	0.56–1.23	0.35	0.90
*MBL2*	rs1800450	0.98	0.71–1.36	0.90	0.98	1.20	0.43–3.34	0.73	1.00
*TNF*	rs1800629	0.99	0.71–1.38	0.95	0.95	0.56	0.16–2.03	0.36	0.79
*IL10*	rs1800896	1.09	0.86–1.38	0.49	0.80	1.03	0.70–1.53	0.87	1.00
***IL6***	**rs2069832**	1.08	0.85–1.38	0.53	0.76	**1.61**	**1.04–2.48**	**0.03**	**0.43**
*ANGPT2*	rs2515475	0.85	0.59–1.38	0.38	0.70	0.66	0.18–2.43	0.52	0.85
*VEGF*	rs3025039	0.84	0.60–1.19	0.34	0.73	0.84	0.24–2.89	0.78	1.00
*IL8*	rs4073	1.13	0.90–1.43	0.30	0.79	1.29	0.87–1.92	0.21	0.90
*EGF*	rs4444903	0.80	0.62–1.03	0.08	0.55	1.33	0.85–2.07	0.21	0.70
*PBEF1*	rs59744560	1.26	0.91–1.74	0.17	0.57	1.08	0.39–3.02	0.89	0.96
*PBEF1*	rs61330082	0.82	0.62–1.07	0.14	0.62	0.99	0.52–1.89	0.99	0.99
*NFE2L2*	rs6721961	1.11	0.77–1.58	0.58	0.69	2.22	0.68–7.25	0.20	1.00

Multivariate logistic regression adjusted for age, sex, APACHE III score, alcohol abuse, smoking status, and history of chronic renal failure and diabetes mellitus.

We evaluated whether we could complete meta-analyses by combining our genotype results with the previously published results but found that the many of the publications lacked adequate genotyping results details. However, we were able to complete meta-analyses for several of the SNPs including in *NAMPT, VEGF, IL8, MBL2* and *TNF*. In unadjusted genotype association meta-analyses using additive modeling, we observed an association between rs59744560 in *NAMPT* and increased risk for ALI (OR 1.33 (95% CI 1.14–1.56) p value <0.0003) and an association between rs61330082 in *NAMPT* and decreased risk for ALI (OR 0.86 (95% CI 0.74–1.0) p value 0.049). We did not observe associations between SNPs in *VEGF, IL8, MBL2* or *TNF* and ALI risk in this meta-analysis. See Table S3.

### Association between SNPs and development of moderate or severe ARDS

We performed a sensitivity analysis excluding subjects with a higher likelihood for phenotypic misclassification as suggested by prior reports [13], [18]. We compared controls (n = 288) without hypoxemic respiratory failure and cases with the more severe lung injury phenotypes, moderate and severe ARDS, n = 126. We identified a nominal association between rs61330082 in *NAMPT* and reduced ARDS susceptibility (OR_adj_ 0.61; 95% confidence interval [CI], 0.40–0.95, *P* = 0.02) which replicates the direction of effect seen previously for this SNP [19]. We observed trends toward increased ARDS risk for homozygous carriers of rs6721961 in *NFE2L2* (OR_adj_ 3.83; 95% confidence interval [CI], 0.93–15.7), and rs2069832 in *IL6* (OR_adj_ 1.91; 95% confidence interval [CI], 0.96–3.40) (Table 5). None of the other SNPs were associated with ARDS risk in this sensitivity analysis.

**Table 5:** Associations with moderate or severe ARDS susceptibility.

		ADDITIVE				RECESSIVE			
	Predictor	Odds Ratio	OR Conf. Interval	P-Value	FDR	Odds Ratio	OR Conf. Interval	P-Value	FDR
*SFTPB*	rs1130866	1.05	0.73–1.51	0.80	0.87	0.85	0.46–1.55	0.59	0.70
*MBL2*	rs1800450	0.86	0.54–1.37	0.52	0.75	0.69	0.17–2.79	0.60	0.64
*TNF*	rs1800629	0.87	0.53–1.43	0.58	0.76	0.49	0.09–2.63	0.40	0.74
*IL10*	rs1800896	1.29	0.92–1.8	0.14	0.94	1.23	0.70–2.16	0.47	0.77
***IL6***	**rs2069832**	1.22	0.85–1.74	0.28	0.90	**1.81**	**0.96–3.40**	**0.068**	**0.44**
*ANGPT2*	rs2515475	0.92	0.56–1.50	0.74	0.87	0.65	0.13–3.15	0.58	0.75
*VEGF*	rs3025039	0.74	0.45–1.23	0.24	1.00	1.01	0.21–4.87	0.99	0.99
*IL8*	rs4073	1.20	0.85–1.68	0.29	0.76	1.57	0.88–2.80	0.13	0.41
*EGF*	rs4444903	0.87	0.61–1.25	0.45	0.74	1.36	0.71–2.60	0.35	0.77
*NAMPT*	rs59744560	1.06	0.65–1.74	0.81	0.81	0.24	0.04–1.67	0.11	0.49
***NAMPT***	**rs61330082**	**0.62**	**0.40–0.95**	**0.02**	**0.30**	0.71	0.24–2.08	0.52	0.75
***NFE2L2***	**rs6721961**	1.27	0.79–2.06	0.33	0.72	**3.83**	**0.94–15.6**	**0.066**	**0.86**

Multivariate logistic regression adjusted for age, sex, APACHE III score, alcohol abuse, smoking status, and history of chronic renal failure and diabetes mellitus. n = 414 (126 with ARDS).

### Associations with mortality and ventilator free-days

Several of the SNPs selected for this study were previously associated with ALI-related outcomes [17], [20], [21]. In secondary analyses, we tested the 12 SNPs for associations with 28 day mortality and VFD among subjects with ALI. Genotype frequencies for survivors and non-survivors of ALI are presented in Table S2. We observed nominal associations between rs4073 in *IL8* and rs1800450 in *MBL2* and decreased VFD in adjusted analyses (Table 6). We also observed nominal associations between rs61330082 in *NAMPT* and rs6721961 in *NFE2L2* and increased risk for mortality when using a recessive model (Table 7). Neither of these associations met our pre-test threshold for significance by FDR. Using genotype data included in published studies, we combined our data and the published data to complete meta-analyses for the SNPs in *TNF* and *MBL2*. In an unadjusted genotype association meta-analysis using recessive modeling, rs1800629 in *TNF* was associated with increased mortality (OR 4.6 (95% CI 1.4–14.9), p value 0.001) and rs1800450 in*MBL2* displayed a trend toward increased risk (OR 3.07 (95% CI 0.99–9.5), p value 0.07). See Table S4.

**Table 6:** Associations with VFDs.

		ADDITIVE			RECESSIVE		
	Predictor	Slope	P-value	FDR	Slope	P-value	FDR
*SFTPB*	rs1130866	0.07	0.94	0.94	0.09	0.96	0.96
***MBL2***	**rs1800450**	−0.43	0.75	0.97	**−10.25**	**0.0149**	**0.19**
*TNF*	rs1800629	−1.00	0.48	1.00	−2.07	0.70	0.91
*IL10*	rs1800896	−0.32	0.75	1.00	−1.03	0.52	0.96
*IL6*	rs2069832	0.78	0.41	1.00	1.03	0.55	0.89
*ANGPT2*	rs2515475	−0.73	0.64	1.00	4.32	0.47	1.00
*VEGF*	rs3025039	−1.53	0.28	1.00	−1.95	0.70	0.83
***IL8***	**rs4073**	−0.70	0.47	1.00	**−3.73**	**0.0196**	**0.13**
*EGF*	rs4444903	−0.82	0.38	1.00	−2.93	0.10	0.43
*NAMPT*	rs59744560	0.46	0.73	1.00	1.11	0.79	0.86
*NAMPT*	rs61330082	−0.22	0.84	0.91	−2.25	0.39	1.00
*NFE2L2*	rs6721961	0.36	0.80	0.95	−3.16	0.50	1.00

Multivariate linear regression adjusted for age, sex, APACHE III score, alcohol abuse, smoking status, and history of chronic renal failure and diabetes mellitus. n = 224.

**Table 7:** Associations with 28-day ALI-related mortality.

		ADDITIVE				RECESSIVE			
	Predictor	Odds Ratio	OR Conf. Interval	P-Value	FDR	Odds Ratio	OR Conf. Interval	P-Value	FDR
*SFTPB*	rs1130866	0.91	0.55–1.50	0.71	1.00	0.73	0.30–1.73	0.46	0.80
*MBL2*	rs1800450	1.02	0.52–2.01	0.96	0.96	1.74	0.27–11.3	0.57	0.76
*TNF*	rs1800629	0.65	0.31–1.34	0.23	0.76	6.00	0.41–87.2	0.17	0.67
*IL10*	rs1800896	0.81	0.48–1.35	0.42	0.78	0.80	0.35–1.83	0.60	0.72
*IL6*	rs2069832	0.96	0.59–1.58	0.88	1.00	0.94	0.39–2.26	0.89	0.97
*ANGPT2*	rs2515475	1.12	0.50–2.5	0.78	1.00				
*VEGF*	rs3025039	1.27	0.62–2.60	0.52	0.84	1.11	0.09–14.5	0.93	0.93
*IL8*	rs4073	1.01	0.62–1.66	0.95	1.00	1.49	0.67–3.31	0.33	0.79
*EGF*	rs4444903	1.36	0.84–2.21	0.21	1.00	1.79	0.75–4.26	0.19	0.58
*NAMPT*	rs59744560	1.46	0.77–2.80	0.25	0.66	2.26	0.32–15.8	0.43	0.85
***NAMPT***	**rs61330082**	1.42	0.82–2.45	0.22	0.94	**4.37**	**1.36–14.1**	**0.016**	**0.20**
***NEF2L2***	**rs6721961**	1.40	0.68–2.88	0.37	0.80	**9.73**	**1.27–74.8**	**0.030**	**0.18**

Multivariate logistic regression adjusted for age, sex, APACHE III score, alcohol abuse, smoking status, and history of chronic renal failure and diabetes mellitus. n = 224 (49 non-survivors at 28 days).

## Discussion

At the time of our study, candidate gene association studies had identified more than 30 genetic variants in over 27 genes that associate with risk for ALI or ALI-related outcomes. These associations provide evidence that genetic factors contribute to ALI and strengthen the link between these specific genes in disease pathogenesis. In this study, we sought to identify robust associations between previously-associated genetic variants and risk for ALI and related outcomes. Using a nested case-control study in a well-phenotyped ICU cohort, we compared SIRS subjects who remained at-risk for ALI to those who developed ALI. Although, in the primary multivariable analyses, the significance levels for the associations with ALI did not survive correction for multiple comparisons, our nominal association results provide some support for prior findings. We observed a nominal association between a SNP in *IL6* (rs2069832, surrogate for rs1800795) and risk for ALI. In analyses restricting case and control definitions to minimize misclassification, we observed a nominal association between rs61330082 in *NAMPT* and reduced risk for moderate and severe ARDS consistent with previous reports [19]. In terms of ALI-related outcomes, we observed nominal associations between rs4073 in *IL8* and rs1800450 in *MBL2* and decreased VFDs. We observed nominal associations between rs61330082 in *NAMPT* and rs6721961 in *NFE2L2* and increased 28-day mortality. None of the associations observed in the secondary analyses survived correction for multiple hypothesis testing.

*IL6* and *IL8* have been extensively studied as important proinflammatory mediators in ALI. Elevated levels of IL-6 and IL-8 are associated with development of ALI, and persistence of elevated levels has been associated with poor outcomes [22]–[24]. rs1800795 is a promoter SNP that results in decreased expression of IL-6, and multiple disease associations have been reported for this SNP [25]. Similarly, rs4073 in *IL8* is a promoter SNP that affects gene transcription and has multiple published disease associations [26]–[28]. Our study employed a larger carefully-phenotyped ICU population as compared to the prior published studies for genetic risk associations between these genes and ALI. The CC genotype of rs1800795 in *IL6* which results in lower promoter activity was previously associated with reduced mortality in ARDS but not with risk for ALI [17]. Nonas et al. observed that a 3-SNP haplotype in *IL6* that included the C allele of rs1800795 was actually associated with reduced risk of ALI [29]. In a subsequent publication, Flores et al. tested 14 SNPs across *IL6* tagging all common linkage disequilibrium bins and found that none of these SNPs demonstrated an association with ALI. However, they did find that carriage of two copies of a 6-SNP haplotype that included the G allele at rs1800795 was associated with an OR of 2.73 (95% CI, 2.39–5.37) for development of ALI [11]. Finally, Sutherland et al. reported that carriage of 2 copies of an *IL6* haplotype clade that included the G allele of rs1800795 was associated with increased risk of death and fewer days alive and free of ALI [30]. Here we now report that a surrogate for rs1800795 (rs2069832, r^2^ = 0.98), is associated with increased risk for ALI and, similar to the studies by Flores and Sutherland, the effect was best modeled assuming a recessive effect. The SNP in *IL8* was previously associated with increased days on mechanical ventilation [20]; in our study, rs4073 in *IL8* was similarly associated with reduced days alive and free of ventilation. These data suggest that common variants in inflammatory cytokines contribute to disease pathogenesis among subjects at risk for or who have ALI.

*NAMPT* (PBEF1) has been associated with lung injury pathogenesis in recent translational and genetic association studies. Pre-B-cell colony-enhancing factor (PBEF) is a cytokine encoded by the gene *NAMPT* and that has been shown to be present at higher concentrations in serum and BALF from patients with ALI [19]. Two studies have reported associations between SNPs in *NAMPT* and development of ALI. Ye et al. identified 11 SNPs in *NAMPT* and, in a comparison between ALI cases and healthy controls, demonstrated that two of the SNPs in the promoter region, T-1001G (rs59744560) and C-1543T (rs61330082) were associated with risk for sepsis and ALI [19]. A second study using a nested case-control design in a larger population replicated the association with the −1001G allele and increased risk for ALI while the −1543T allele was associated with decreased risk for ARDS [9]. In our study, we observed the same effect of reduced risk of ALI for carriers of the −1543T genotype in sensitivity analyses restricting to cases with ARDS and controls without hypoxemia. On the other hand, we did not replicate the risk association for the −1001G SNP (rs59744560) in our primary adjusted analysis. In a post hoc meta-analysis combining our data and published genotype data for rs59744560, we did observe a strongly significant association with risk for ALI. Taken together, these findings support a role for *NAMPT* in ALI pathogenesis and suggest that *NAMPT* promoter variants are associated with risk for disease development.

*NFE2L2* is a gene that has been identified as a potential mediator of hyperoxia-induced lung damage via linkage analysis and positional cloning in mice [31]. *NFE2L2* is a transcription factor that targets antioxidant response elements (AREs) leading to gene regulation that is protective in the setting of oxidative stress. Resequencing of *NFE2L2* identified a variant (rs6721961) that resulted in diminished promoter activity in an *in vitro* transfection experiment [8]. The authors also reported that rs6721961 was associated with increased risk for development of trauma-associated acute lung injury. We observed a trend for association of this variant with increased risk for ALI among subjects with SIRS, with a stronger trend in the sensitivity analysis with ARDS as the outcome. Our analysis resulted in a strong odds ratio for increased risk for developing ALI similar to the prior report [8].

Misclassification of ALI, particularly the inclusion of subjects without true ALI among cases, can lead to significant reductions in statistical power to detect true genetic associations [32]. For example, a recent study found that removal of subjects with equivocal CXR results from a set of patients classified with ALI resulted in an improvement in the strength of associations observed [18]. We undertook a sensitivity analysis to determine if we would observe stronger associations following reduction of potential misclassification of ALI cases and at-risk controls. We restricted cases to only those ALI cases qualifying for ARDS (PF<200) while also restricting control subjects to those who did not have hypoxemia. This approach reduced the analysis sample size but resulted in associations with effects that were farther from the null for the SNPs in *IL6*, *NAMPT* and *NFE2L2*. While these findings did not achieve statistical significance after correction for type I error they provide support for the contention that phenotypic misclassification is a major issue reducing power and precision in case-control studies of ALI.

Overall, our study failed to demonstrate robust associations between the SNPs studied and risk for ALI and related outcomes. Reasons for lack of validation include inadequate statistical power, misclassification of subject phenotypes, sample population differences compared to the prior studies, population substructure, and absence of true association with ALI. As power was limiting to identify small effect sizes, especially in the recessive model, we cannot exclude the possibility that we failed to observe true associations between the SNPs and ALI susceptibility and related outcomes. A limited analysis on 67 genotypes demonstrated that subjects from our cohort clustered with HapMap CEU and TSI subjects suggesting that there was not obvious evidence for cryptic admixture. However, more subtle differences in population substructure could have also have affected our ability to detect true associations. Finally, most of the SNPs included in this study have not been definitively shown to have functional significance and may merely serve as markers for causal variants. Future work will require more dense genotyping or sequencing at these loci to gain further insight into potential mechanisms.

This study used a well-phenotyped cohort of ICU subjects with SIRS and a nested-case control design to replicate ALI risk associations for several previously identified genetic variants. We observed nominal associations for between SNPS in *IL6* and *NAMPT*, and risk for ALI and ARDS, but these associations failed to meet significance after adjusting for multiple comparisons. Nominal associations were also identified between SNPs in *IL8, MBL2, NAMPT* and *NFE2L2* and ALI severity outcomes, namely VFDs and 28-day mortality. The failure to replicate the majority of ALI risk and outcome associations with statistical significance emphasizes the challenges of genetic association studies in critical care populations including sample size and disease classification. Our results also highlight the importance of independent confirmatory studies of associations with ALI susceptibility and related outcomes in order to support a better understanding of ALI pathogenesis and prognosis.

## Supporting Information

Figure S1 **Genotype principal component analysis plot demonstrating overlap of our subjects with HapMap ethnic populations.** Population stratification was assessed by PCA for 67 shared genotypes among our subjects and HapMap 3 subjects [12]. Our subjects overlapped with CEU and TSI HapMap subjects but separated from subjects of African or Asian ethnicity when plotting eigenvalues 1 versus 2.
(TIF)

Figure S2 **Plot of power estimates based on minor allele frequency and genotype effect size.** Using CaTS [39], we generated results for estimated statistical power (1-b>0.8) to detect additive and recessive model associations over varying allele frequencies and genotype relative risks, using our known sample size and case frequency and an alpha error rate of 0.05. Each line represents a genotype relative risk: A, power estimates for the additive model for genotype relative risks of 1.25, 1.5, 1.75 and 2; B, power estimates for the recessive model for genotype relative risks of 1.5, 2, 2.5 and 3.
(TIF)

Table S1 **Genetic variants associated with acute lung injury and associated outcomes in peer reviewed publications.**
(DOCX)

Table S2 **Genotype frequencies and unadjusted frequency analysis for ALI survivors vs. non-survivors at 28 days.**
(DOCX)

Table S3 **Meta-analysis for ALI risk: genotype frequencies used in meta-analysis calculations.** Results for Fisher exact test.
(DOCX)

Table S4 **Meta-analysis for ALI-related mortality: genotype frequencies used in meta-analysis calculations.** Results for Fisher exact test.
(DOCX)
