# Supplementary figures and images for: Inflammation and Immune-Related Candidate Gene Associations with Acute Lung Injury Susceptibility and Severity: A Validation Study
Source: PLoS One. 2012 Dec 14;7(12):e51104. doi: 10.1371/journal.pone.0051104 (PMC3522667; doi:10.1371/journal.pone.0051104)

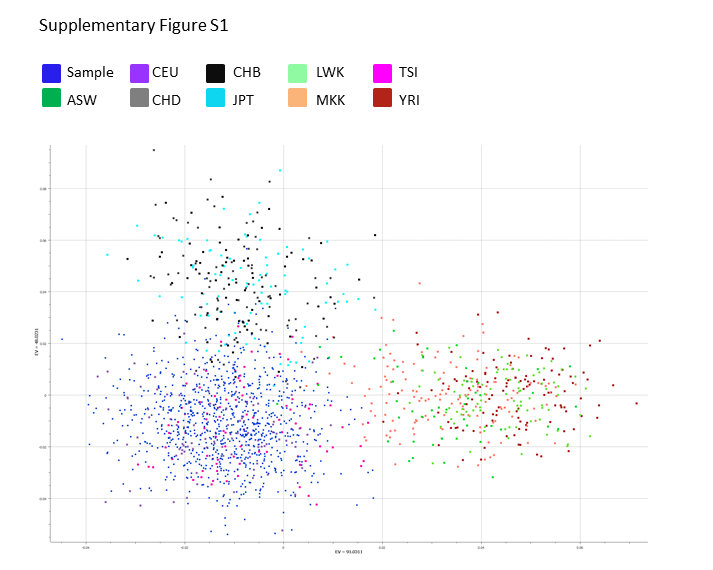

Supplement: Figure S1 — Genotype principal component analysis plot demonstrating overlap of our subjects with HapMap ethnic populations. Population stratification was assessed by PCA for 67 shared genotypes among our subjects and HapMap 3 subjects [12]. Our subjects overlapped with CEU and TSI HapMap subjects but separated from subjects of African or Asian ethnicity when plotting eigenvalues 1 versus 2. (TIF) [file pone.0051104.s001.tif]

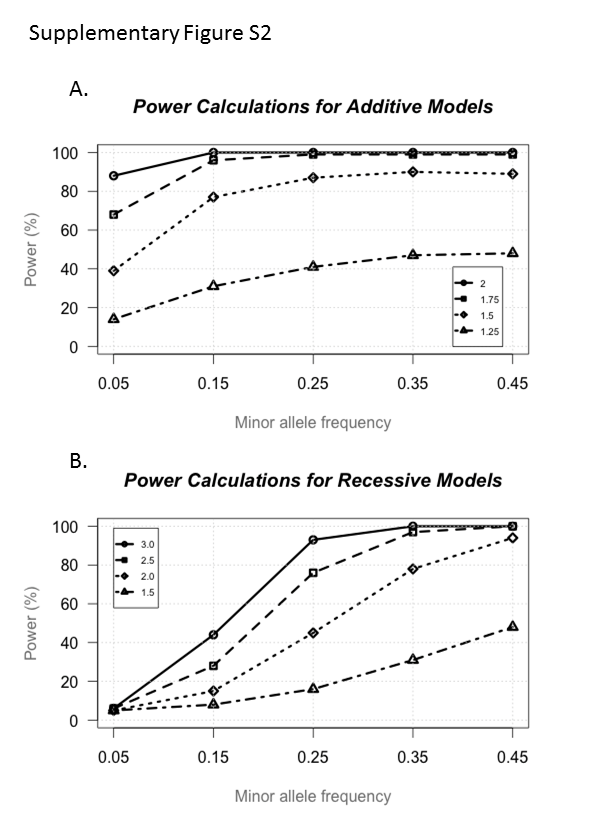

Supplement: Figure S2 — Plot of power estimates based on minor allele frequency and genotype effect size. Using CaTS [39], we generated results for estimated statistical power (1-b>0.8) to detect additive and recessive model associations over varying allele frequencies and genotype relative risks, using our known sample size and case frequency and an alpha error rate of 0.05. Each line represents a genotype relative risk: A, power estimates for the additive model for genotype relative risks of 1.25, 1.5, 1.75 and 2; B, power estimates for the recessive model for genotype relative risks of 1.5, 2, 2.5 and 3. (TIF) [file pone.0051104.s002.tif]
