# Supplementary material for: Inflammation and Immune-Related Candidate Gene Associations with Acute Lung Injury Susceptibility and Severity: A Validation Study
Source: PLoS One. 2012 Dec 14;7(12):e51104. doi: 10.1371/journal.pone.0051104 (PMC3522667; doi:10.1371/journal.pone.0051104)
Supplement: Table S2 — Genotype frequencies and unadjusted frequency analysis for ALI survivors vs. non-survivors at 28 days. (DOCX) [file pone.0051104.s004.docx]

Supplementary Table S2.

Genotype frequencies and unadjusted frequency analysis for ALI survivors vs. non-survivors at 28 days.

| Gene | dbSNP rs# | Genotypes in ALI survivors | (n=169) |  | χ2 p-value for HWE in survivors | Genotypes in ALI non-survivors | (n=55) |  | Fisher’s Exact test for genotype freq. (p-value)^a^ |
| --- | --- | --- | --- | --- | --- | --- | --- | --- | --- |
| *SFTPB* | rs1130866 | TT 44 | TC 87 | CC 38 | 0.69 | TT 13 | TC 29 | CC 13 | 0.96 |
| *MBL2* | rs1800450 | CC 122 | CT 43 | TT 4 | 0.93 | CC 40 | CT 13 | TT 2 | 0.81 |
| *TNF* | rs1800629 | GG 117 | GA 51 | AA 1 | 0.07 | GG 43 | GA 9 | AA 3 | **0.01** |
| *IL10* | rs1800896 | TT 42 | TC 89 | CC 38 | 0.48 | TT 17 | TC 25 | CC 13 | 0.57 |
| *IL6* | rs2069832 | GG 61 | GA 72 | AA 36 | 0.09 | GG 17 | GA 27 | AA 11 | 0.70 |
| *ANGPT2* | rs2515475 | CC 137 | CT 29 | TT 3 | 0.32 | CC 41 | CT 41 | TT 1 | 0.53 |
| *VEGF* | rs3025039 | CC 130 | CT 36 | TT 3 | 0.78 | CC 38 | CT 16 | TT 1 | 0.45 |
| *IL8* | rs4073 | TT 46 | TA 85 | AA 38 | 0.92 | TT 15 | TA 23 | AA 17 | 0.42 |
| *EGF* | rs4444903 | AA 73 | AG 69 | GG 27 | 0.12 | AA 18 | AG 24 | GG 13 | 0.27 |
| *NAMPT* | rs59744560 | GG 121 | GT 44 | TT 4 | 1.00 | GG 38 | GT 15 | TT 2 | 0.77 |
| *NAMPT* | rs61330082 | CC 108 | CT 52 | TT 9 | 0.41 | CC 33 | CT 15 | TT 3 | 0.21 |
| *NFE2L2* | rs6721961 | GG 131 | GT 36 | TT 2 | 0.79 | GG 44 | GT 8 | TT 3 | 0.10 |

HWE Hardy-Weinberg equilibrium; ^a^ p value for the Fisher’s exact test comparing genotypes for at ALI survivors and non-survivors at 28 days
