# Supplementary material for: Inflammation and Immune-Related Candidate Gene Associations with Acute Lung Injury Susceptibility and Severity: A Validation Study
Source: PLoS One. 2012 Dec 14;7(12):e51104. doi: 10.1371/journal.pone.0051104 (PMC3522667; doi:10.1371/journal.pone.0051104)
Supplement: Table S3 — Meta-analysis for ALI risk: genotype frequencies used in meta-analysis calculations. Results for Fisher exact test. (DOCX) [file pone.0051104.s005.docx]

Supplementary Table S3.

Meta-analysis for ALI risk: Genotype frequencies

| Gene | dbSNP rs# | Genotypes in at risk controls |  |  | Genotypes in ALI cases |  |  | Fisher’s Exact test for genotype freq. (p-value)^a^ | Data sources |
| --- | --- | --- | --- | --- | --- | --- | --- | --- | --- |
| *SFTPB* | rs1130866 | † |  |  |  |  |  |  |  |
| *MBL2* | rs1800450 | CC 689 | CT 262 | TT 17 | CC 313 | CT 110 | TT 13 | 0.3 | [15] |
| *TNF* | rs1800629 | GG 456 | GA 155 | AA 15 | GG 233 | GA 78 | AA 9 | 0.9 | [34] |
| *IL10* | rs1800896 | † |  |  |  |  |  |  |  |
| *IL6* | rs2069832 | † |  |  |  |  |  |  |  |
| *ANGPT2* | rs2515475 | † |  |  |  |  |  |  |  |
| *VEGF* | rs3025039 | CC 464 | CT &TT 165 | | CC 243 | CT & TT 93 | | 0.2 | [31] |
| *IL8* | rs4073 | TT 183 | TA 282 | AA 132 | TT 71 | TA 119 | AA 57 | 0.85 | [14] |
| *EGF* | rs4444903 | † |  |  |  |  |  |  |  |
| *NAMPT* | rs59744560 | GG 935 | GT 397 | TT 63 | GG 385 | GT 249 | TT 34 | 0.0001 | [25,26] |
| *NAMPT* | rs61330082 | CC 765 | CT 509 | TT 91 | CC 419 | CT 214 | TT 41 | 0.077 | [25,26] |
| *NFE2L2* | rs6721961 | † |  |  |  |  |  |  |  |

† Insufficient data in published reports

*NAMPT* rs59744560: in additive modeling, OR for ALI risk is 1.33 (95% CI 1.14-1.56) p value <0.0003

*NAMPT* rs61330082: in additive modeling, OR for ALI risk is OR 0.86 (95% CI 0.74-1.0) p value 0.049
