# Supplementary material for: Inflammation and Immune-Related Candidate Gene Associations with Acute Lung Injury Susceptibility and Severity: A Validation Study
Source: PLoS One. 2012 Dec 14;7(12):e51104. doi: 10.1371/journal.pone.0051104 (PMC3522667; doi:10.1371/journal.pone.0051104)
Supplement: Table S4 — Meta-analysis for ALI-related mortality: genotype frequencies used in meta-analysis calculations. Results for Fisher exact test. (DOCX) [file pone.0051104.s006.docx]

Supplementary Table S4.

Meta-analysis for ALI-related mortality: Genotype frequencies

| Gene | dbSNP rs# | Genotypes in ALI Survivors at 28 days |  |  | Genotypes in ALI non-survivors at 28 days |  |  | Fisher’s Exact test for genotype freq. (p-value)^a^ | Data sources |
| --- | --- | --- | --- | --- | --- | --- | --- | --- | --- |
| *SFTPB* | rs1130866 | † |  |  |  |  |  |  |  |
| *MBL2* | rs1800450 | CC 200 | CT 78 | TT 5 | CC 113 | CT 32 | TT 8 | 0.053 | [15] |
| *TNF* | rs1800629 | GG 197 | GA 69 | AA 4 | GG 108 | GA 37 | AA 10 | 0.02 | [34] |
| *IL10* | rs1800896 | † |  |  |  |  |  |  |  |
| *IL6* | rs2069832 | † |  |  |  |  |  |  |  |
| *ANGPT2* | rs2515475 | † |  |  |  |  |  |  |  |
| *VEGF* | rs3025039 | † |  | |  |  | |  |  |
| *IL8* | rs4073 | † |  |  |  |  |  |  |  |
| *EGF* | rs4444903 | † |  |  |  |  |  |  |  |
| *NAMPT* | rs59744560 | † |  |  |  |  |  |  |  |
| *NAMPT* | rs61330082 | † |  |  |  |  |  |  |  |
| *NFE2L2* | rs6721961 | † |  |  |  |  |  |  |  |

† Insufficient data in published reports

MBL2: in recessive modeling, OR for mortality 3.07 (95% CI 0.99 - 9.5)

TNF: in recessive modeling, OR for mortality 4.6 (95% CI 1.4 - 14.9)
