# Supplementary material for: Inflammation and Immune-Related Candidate Gene Associations with Acute Lung Injury Susceptibility and Severity: A Validation Study
Source: PLoS One. 2012 Dec 14;7(12):e51104. doi: 10.1371/journal.pone.0051104 (PMC3522667; doi:10.1371/journal.pone.0051104)
Supplement: Table S1 — Genetic variants associated with acute lung injury and associated outcomes in peer reviewed publications. (DOCX) [file pone.0051104.s003.docx]

Supplementary Table S1.

Genetic variants associated with acute lung injury and associated outcomes in peer reviewed publications.

| Gene | Variant | dbSNP | Ref |
| --- | --- | --- | --- |
| *ACE* | deletion/insertion in intron 16 |  | [1][2] |
| *ANGPT2* | Intronic C/T | rs2515475 | [3] |
| *EGF* | A61G | rs4444903 | [4] |
| *F5* | Arg506Gln | rs6025 | [5] |
| *FAS* | T-9019A | rs17447091 | [6] |
| *FTL* | 3 SNP Haplotype |  | [7] |
| *HMOX1* | 5 SNP Haplotype |  | [7] |
| *IL10* | G-1082A | rs1800896 | [8][9] |
| *IL6* | G-174C | rs1800795 | [10] |
| *IL6* | Haplotype: -174C, 1753C, 2954G |  | [11] |
| *IL6* | 3 SNP haplotype, 6 SNP haplotype |  | [12] [13] |
| *IL8* | A-251T | rs4073 | [14] |
| *MBL2* | Codon 54 | rs1800450 | [15] |
| *MIF* | 8 SNP Haplotype |  | [16] |
| *MYLK* | 25-28 SNPs Haplotypes |  | [17][18] |
| *NFKB1* | -94 in/del ATTG |  | [19] |
| *NFKBIA* | A-881G, C-826T, C-297T |  | [20] |
| *NQO1* | A-1221C | rs689455 | [21] |
| *NFE2L2* | C-617A | rs6721961 | [22] |
| *PAI1* | 4G/5G | rs1799768 | [23][24] |
| *NAMPT* | T1001G, C1543T | rs59744560, rs61330082 | [25][26] |
| *PI3* | T34P | rs2664581 | [27] |
| *PLAU* | 6 SNP Haplotype |  | [28] |
| *SFTPB* | Intron 4 repeats |  | [29] |
| *SFTPB* | T1580C | rs1130866 | [30][31] |
| *SOD3* | 28 SNP Haplotype |  | [32] |
| *TLR1* | -7202A/G | rs5743551 | [33] |
| *TNF* | G-308A | rs1800629 | [34] |
| *LTA* | TNFB2 Nco I polymorphism |  | [34] |
| *VEGF* | C936T | rs3025039 | [35] |
| *VEGF* | Haplotype: C-460T, C405G, C936T |  | [36] |

References

1.	Adamzik M, Frey U, Sixt S, Knemeyer L, Beiderlinden M, et al. (2007) ACE I/D but not AGT (-6)A/G polymorphism is a risk factor for mortality in ARDS. Eur Respir J 29: 482–488. doi:10.1183/09031936.00046106.

2.	Marshall RP, Webb S, Bellingan GJ, Montgomery HE, Chaudhari B, et al. (2002) Angiotensin converting enzyme insertion/deletion polymorphism is associated with susceptibility and outcome in acute respiratory distress syndrome. Am J Respir Crit Care Med 166: 646–650.

3.	Su L, Zhai R, Sheu C-C, Gallagher DC, Gong MN, et al. (2009) Genetic variants in the angiopoietin-2 gene are associated with increased risk of ARDS. Intensive Care Med 35: 1024–1030. doi:10.1007/s00134-009-1413-8.

4.	Sheu CC, Zhai R, Su L, Tejera P, Gong MN, et al. (2009) Sex-specific association of epidermal growth factor gene polymorphisms with acute respiratory distress syndrome. Eur Respir J 33: 543–550. doi:10.1183/09031936.00091308.

5.	Adamzik M, Frey UH, Riemann K, Sixt S, Lehmann N, et al. (2008) Factor V Leiden mutation is associated with improved 30-day survival in patients with acute respiratory distress syndrome. Crit Care Med 36: 1776–1779. doi:10.1097/CCM.0b013e318174373d.

6.	Glavan BJ, Holden TD, Goss CH, Black RA, Neff MJ, et al. (2011) Genetic variation in the FAS gene and associations with acute lung injury. Am J Respir Crit Care Med 183: 356–363. doi:10.1164/rccm.201003-0351OC.

7.	Lagan AL, Quinlan GJ, Mumby S, Melley DD, Goldstraw P, et al. (2008) Variation in iron homeostasis genes between patients with ARDS and healthy control subjects. Chest 133: 1302–1311. doi:10.1378/chest.07-1117.

8.	Gong MN, Thompson BT, Williams PL, Zhou W, Wang MZ, et al. (2006) Interleukin-10 polymorphism in position -1082 and acute respiratory distress syndrome. Eur Respir J 27: 674–681. doi:10.1183/09031936.06.00046405.

9.	Schroeder O, Schulte K-M, Schroeder J, Ekkernkamp A, Laun RA (2008) The -1082 interleukin-10 polymorphism is associated with acute respiratory failure after major trauma: a prospective cohort study. Surgery 143: 233–242. doi:10.1016/j.surg.2007.07.040.

10.	Marshall RP, Webb S, Hill MR, Humphries SE, Laurent GJ (2002) Genetic polymorphisms associated with susceptibility and outcome in ARDS. Chest 121: 68S–69S.

11.	Sutherland AM, Walley KR, Manocha S, Russell JA (2005) The association of interleukin 6 haplotype clades with mortality in critically ill adults. Arch Intern Med 165: 75–82. doi:10.1001/archinte.165.1.75.

12.	Nonas SA, Finigan JH, Gao L, Garcia JGN (2005) Functional genomic insights into acute lung injury: role of ventilators and mechanical stress. Proc Am Thorac Soc 2: 188–194. doi:10.1513/pats.200501-005AC.

13.	Flores C, Ma S-F, Maresso K, Wade MS, Villar J, et al. (2008) IL6 gene-wide haplotype is associated with susceptibility to acute lung injury. Transl Res 152: 11–17. doi:10.1016/j.trsl.2008.05.006.

14.	Hildebrand F, Stuhrmann M, van Griensven M, Meier S, Hasenkamp S, et al. (2007) Association of IL-8-251A/T polymorphism with incidence of Acute Respiratory Distress Syndrome (ARDS) and IL-8 synthesis after multiple trauma. Cytokine 37: 192–199. doi:10.1016/j.cyto.2007.03.008.

15.	Gong MN, Zhou W, Williams PL, Thompson BT, Pothier L, et al. (2007) Polymorphisms in the mannose binding lectin-2 gene and acute respiratory distress syndrome. Crit Care Med 35: 48–56. doi:10.1097/01.CCM.0000251132.10689.F3.

16.	Gao L, Flores C, Fan-Ma S, Miller EJ, Moitra J, et al. (2007) Macrophage migration inhibitory factor in acute lung injury: expression, biomarker, and associations. Transl Res 150: 18–29. doi:10.1016/j.trsl.2007.02.007.

17.	Gao L, Grant A, Halder I, Brower R, Sevransky J, et al. (2006) Novel polymorphisms in the myosin light chain kinase gene confer risk for acute lung injury. Am J Respir Cell Mol Biol 34: 487–495. doi:10.1165/rcmb.2005-0404OC.

18.	Christie JD, Ma S-F, Aplenc R, Li M, Lanken PN, et al. (2008) Variation in the myosin light chain kinase gene is associated with development of acute lung injury after major trauma. Crit Care Med 36: 2794–2800.

19.	Adamzik M, Frey UH, Rieman K, Sixt S, Beiderlinden M, et al. (2007) Insertion/deletion polymorphism in the promoter of NFKB1 influences severity but not mortality of acute respiratory distress syndrome. Intensive Care Med 33: 1199–1203. doi:10.1007/s00134-007-0649-4.

20.	Zhai R, Zhou W, Gong MN, Thompson BT, Su L, et al. (2007) Inhibitor kappaB-alpha haplotype GTC is associated with susceptibility to acute respiratory distress syndrome in Caucasians. Crit Care Med 35: 893–898. doi:10.1097/01.CCM.0000256845.92640.38.

21.	Reddy AJ, Christie JD, Aplenc R, Fuchs B, Lanken PN, et al. (2009) Association of human NAD(P)H:quinone oxidoreductase 1 (NQO1) polymorphism with development of acute lung injury. J Cell Mol Med 13: 1784–1791. doi:10.1111/j.1582-4934.2008.00581.x.

22.	Marzec JM, Christie JD, Reddy SP, Jedlicka AE, Vuong H, et al. (2007) Functional polymorphisms in the transcription factor NRF2 in humans increase the risk of acute lung injury. FASEB J 21: 2237–2246. doi:10.1096/fj.06-7759com.

23.	Madách K, Aladzsity I, Szilágyi A, Fust G, Gál J, et al. (2010) 4G/5G polymorphism of PAI-1 gene is associated with multiple organ dysfunction and septic shock in pneumonia induced severe sepsis: prospective, observational, genetic study. Crit Care 14: R79. doi:10.1186/cc8992.

24.	Sapru A, Curley MAQ, Brady S, Matthay MA, Flori H (2010) Elevated PAI-1 is associated with poor clinical outcomes in pediatric patients with acute lung injury. Intensive Care Med 36: 157–163. doi:10.1007/s00134-009-1690-2.

25.	Ye SQ, Simon BA, Maloney JP, Zambelli-Weiner A, Gao L, et al. (2005) Pre-B-cell colony-enhancing factor as a potential novel biomarker in acute lung injury. Am J Respir Crit Care Med 171: 361–370. doi:10.1164/rccm.200404-563OC.

26.	Bajwa EK, Yu C-L, Gong MN, Thompson BT, Christiani DC (2007) Pre-B-cell colony-enhancing factor gene polymorphisms and risk of acute respiratory distress syndrome. Crit Care Med 35: 1290–1295. doi:10.1097/01.CCM.0000260243.22758.4F.

27.	Tejera P, Wang Z, Zhai R, Su L, Sheu C-C, et al. (2009) Genetic polymorphisms of peptidase inhibitor 3 (elafin) are associated with acute respiratory distress syndrome. Am J Respir Cell Mol Biol 41: 696–704. doi:10.1165/rcmb.2008-0410OC.

28.	Arcaroli J, Sankoff J, Liu N, Allison DB, Maloney J, et al. (2008) Association between urokinase haplotypes and outcome from infection-associated acute lung injury. Intensive Care Med 34: 300–307. doi:10.1007/s00134-007-0930-6.

29.	Gong MN, Wei Z, Xu L-L, Miller DP, Thompson BT, et al. (2004) Polymorphism in the surfactant protein-B gene, gender, and the risk of direct pulmonary injury and ARDS. Chest 125: 203–211.

30.	Lin Z, Pearson C, Chinchilli V, Pietschmann SM, Luo J, et al. (2000) Polymorphisms of human SP-A, SP-B, and SP-D genes: association of SP-B Thr131Ile with ARDS. Clin Genet 58: 181–191.

31.	Quasney MW, Waterer GW, Dahmer MK, Kron GK, Zhang Q, et al. (2004) Association between surfactant protein B + 1580 polymorphism and the risk of respiratory failure in adults with community-acquired pneumonia. Crit Care Med 32: 1115–1119.

32.	Arcaroli JJ, Hokanson JE, Abraham E, Geraci M, Murphy JR, et al. (2009) Extracellular superoxide dismutase haplotypes are associated with acute lung injury and mortality. Am J Respir Crit Care Med 179: 105–112. doi:10.1164/rccm.200710-1566OC.

33.	Wurfel MM, Gordon AC, Holden TD, Radella F, Strout J, et al. (2008) Toll-like receptor 1 polymorphisms affect innate immune responses and outcomes in sepsis. Am J Respir Crit Care Med 178: 710–720. doi:10.1164/rccm.200803-462OC.

34.	Gong MN, Zhou W, Williams PL, Thompson BT, Pothier L, et al. (2005) -308GA and TNFB polymorphisms in acute respiratory distress syndrome. Eur Respir J 26: 382–389. doi:10.1183/09031936.05.00000505.

35.	Medford ARL, Keen LJ, Bidwell JL, Millar AB (2005) Vascular endothelial growth factor gene polymorphism and acute respiratory distress syndrome. Thorax 60: 244–248. doi:10.1136/thx.2004.034785.

36.	Zhai R, Gong MN, Zhou W, Thompson TB, Kraft P, et al. (2007) Genotypes and haplotypes of the VEGF gene are associated with higher mortality and lower VEGF plasma levels in patients with ARDS. Thorax 62: 718–722. doi:10.1136/thx.2006.069393.
